# The pig transport network in Switzerland: Structure, patterns, and implications for the transmission of infectious diseases between animal holdings

**DOI:** 10.1371/journal.pone.0217974

**Published:** 2019-05-31

**Authors:** Martin Sterchi, Céline Faverjon, Cristina Sarasua, Maria Elena Vargas, John Berezowski, Abraham Bernstein, Rolf Grütter, Heiko Nathues

**Affiliations:** 1 Department of Informatics, University of Zurich, Zurich, Switzerland; 2 Swiss Federal Research Institute WSL, Birmensdorf, Switzerland; 3 School of Business, University of Applied Sciences and Arts Northwestern Switzerland FHNW, Olten, Switzerland; 4 Veterinary Public Health Institute, Vetsuisse Faculty, University of Bern, Bern, Switzerland; 5 Clinic for Swine, Vetsuisse Faculty, University of Bern, Bern, Switzerland; Frankfurt Institute for Advanced Studies, GERMANY

## Abstract

The topology of animal transport networks contributes substantially to how fast and to what extent a disease can transmit between animal holdings. Therefore, public authorities in many countries mandate livestock holdings to report all movements of animals. However, the reported data often does not contain information about the exact sequence of transports, making it impossible to assess the effect of truck sharing and truck contamination on disease transmission. The aim of this study was to analyze the topology of the Swiss pig transport network by means of social network analysis and to assess the implications for disease transmission between animal holdings. In particular, we studied how additional information about transport sequences changes the topology of the contact network. The study is based on the official animal movement database in Switzerland and a sample of transport data from one transport company. The results show that the Swiss pig transport network is highly fragmented, which mitigates the risk of a large-scale disease outbreak. By considering the time sequence of transports, we found that even in the worst case, only 0.34% of all farm-pairs were connected within one month. However, both network connectivity and individual connectedness of farms increased if truck sharing and especially truck contamination were considered. Therefore, the extent to which a disease may be transmitted between animal holdings may be underestimated if we only consider data from the official animal movement database. Our results highlight the need for a comprehensive analysis of contacts between farms that includes indirect contacts due to truck sharing and contamination. As the nature of animal transport networks is inherently temporal, we strongly suggest the use of temporal network measures in order to evaluate individual and overall risk of disease transmission through animal transportation.

## Introduction

Animal transports are widely seen as one of the main factors contributing to the transmission of infectious diseases in animal populations [1]. This has led public authorities in many countries and supranational bodies such as the EU to collect animal movement data. In the last decade, there has been a growing interest in analyzing such data using social network analysis in order to better understand interrelationships between animal holdings (i.e., farms, slaughterhouses, etc.) and assess their impact on disease transmission [2]. The first attempts at modelling animal transport networks with social network analysis were static network models [3,4]. The importance of dynamic (or temporal) network analysis has only recently been established [5–7]. Animal transport networks are inherently temporal, as a link between two holdings only exists at the time of the transport. For the analysis of the transmission of a disease, this consideration is crucial, as links in temporal networks are not necessarily transitive. In other words, a pathogen may only be passed from farm A to farm C via farm B if the corresponding transports between those three farms are sequential in time.

Much of the recent literature on animal transport networks pays particular attention to the overall connectivity of the network and/or to the individual connectivity or centrality of nodes in the network [8–16]. Several researchers have used the overall connectivity of the network as a measure of the potential epidemic size of infectious diseases [8]. The identification of highly central nodes, so called hubs, is considered crucial for designing control and surveillance programs [9] as they constitute so called “super-spreaders” [17]. More recent work has increasingly focused on temporal network measures and has shown that static network measures may overestimate the potential epidemic size or underestimate the individual farm risk [8,9,14,16].

For social network analysis to be most effective for disease control and surveillance, the topology of the network needs to represent the direct and indirect contacts among animals, with the highest accuracy possible. Disease transmission is not restricted to movements of infected animals from one farm to another. A specific animal transport between two farms is often part of a sequence of transports by the same truck, which leads to at least two other, indirect mechanisms for disease transmission. First, animals from different farms may be transported together in the same truck even if they do not end up at the same arrival holding. Second, a truck may be contaminated from a previous transport and upon loading animals at a new farm, may indirectly transmit a pathogen to the new farm [18–20]. The necessity of analyzing and modelling the actual transport sequences has been asserted in the early stages of epidemiological network analysis [4]. However, the exact transport sequences are usually only available from transport companies which record all load and unload operations to manage efficiency. Most previous studies have been restricted to incomplete data, often collected by public authorities, and have neglected considering indirect ways of disease transmission during animal transports. The possibility of analyzing such data has been demonstrated in the French pig industry [10,15], and in a regional study in Canada [13]. These studies show that the aforementioned indirect ways of disease transmission connect holdings that may not be connected if only direct contacts due to animal exchanges are considered. However, whether their findings can be generalized to different production systems remains open, especially when the structure of animal production networks differs considerably between countries.

In Switzerland, the pig production system differs greatly from other countries with large and vertically integrated pork production systems. Swiss pig production is organized in a hierarchical network, highly decentralized, and made up of many small, independent farms. One of the particularities of the Swiss system is the so called "arbeitsteilige Ferkelproduktion" which corresponds to a cyclical process comparable to sow pool systems in Scandinavian countries where sows are bred in a specialized breeding center, then moved to farrowing farms, after which they return to a breeding center which might be different from the initial one. Riklin [21] reports that a large proportion of Swiss fattening farms work with multiple piglet producers leading to a large number of contacts between pigs which has the potential to increase the risk of disease transmission within the production chain. Moreover, Swiss regulations for cleaning and disinfection of trucks (Art. 163, Tierschutzverordnung [22]) are not as strict as European Union regulations. For example, it is not mandatory to disinfect the truck after a transport. This lack of biosecurity measures increases the risk of transmission for several infectious diseases that are transmitted by the fecal-oral route such as salmonella infections, swine dysentery, etc. [18]. The atypical structure of the Swiss pig production system makes comparisons with transport networks in other countries difficult. To date, little has been published about the importance of the structure of the Swiss pig production system for disease control and surveillance. This may also be due to the fact that outbreaks of infectious diseases in Switzerland are rare. One of the more recent outbreaks concerned porcine reproductive and respiratory syndrome virus (PRRSV) and dates back to 2012 [23].

The Federal Food Safety and Veterinary Office (FSVO) records and stores data from pig movements in the animal movement database (AMD) [24]. However, the exact transport sequences are not recorded in these data and hence, the true contact network between Swiss pig farms remains widely unknown. Nonetheless, it is possible to get a partial picture of indirect disease transmission by considering data of transport companies. The main goal of this paper was to assess the effect of additionally considering such transport data provided by a Swiss transport company on network measures relevant for disease transmission. We first described the Swiss pig transport network using data provided by the public authorities (AMD) focusing on temporal network measures. Based on a sample of transport data from the transport company, we then examined if and how the additional information about the sequence of transports changed the connectivity of the network and the node centrality measures. Finally, we discussed the results and their implications for the Swiss pig industry.

## Materials and methods

### Data

**Fig 1:**
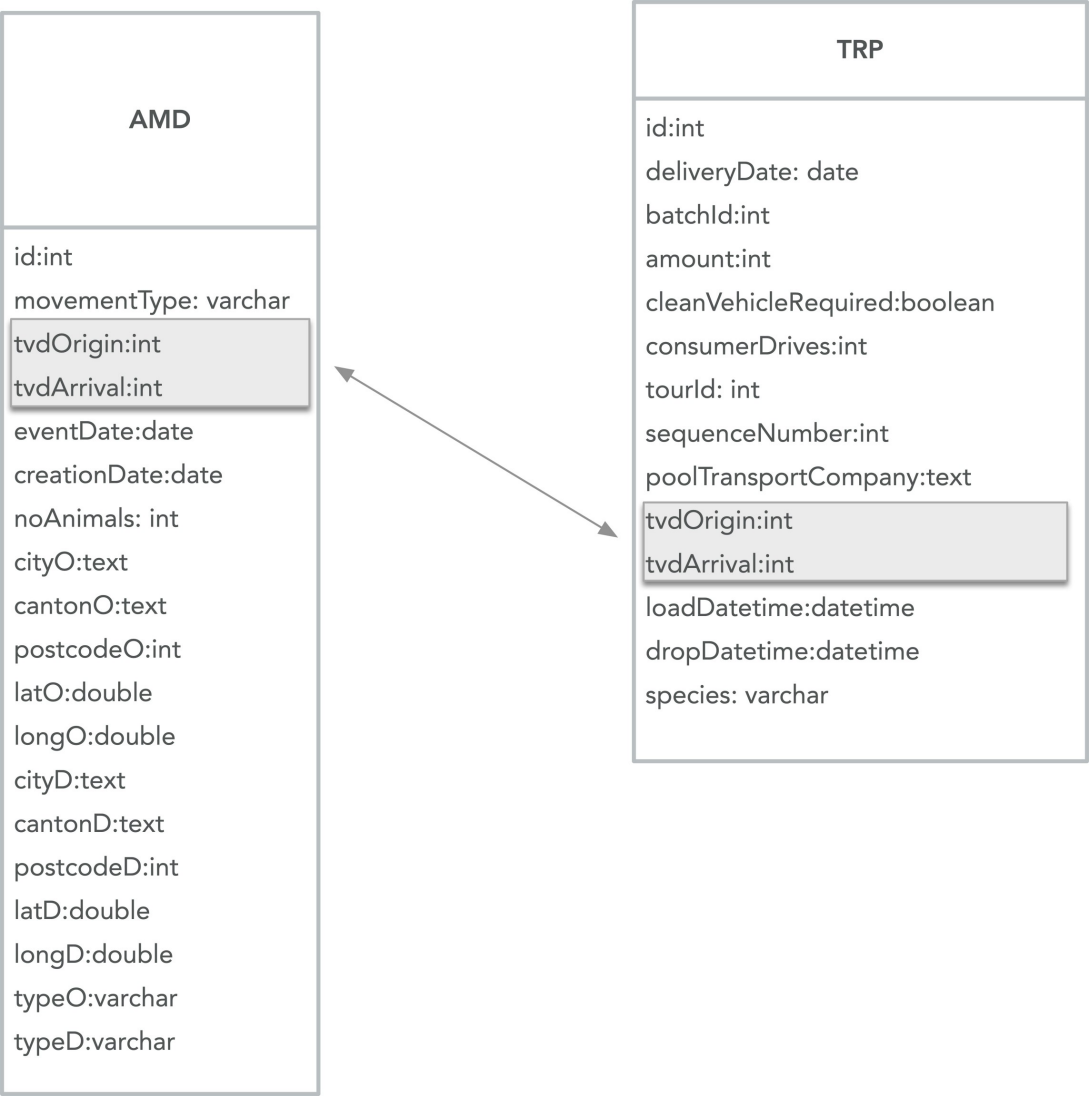
**Graphical representation of the raw data we collected from AMD (on the left) and TRP (on the right)**. The matching of movements is based on the attributes tvdOrigin (integer) and tvdArrival (integer) in both data sets.

This study is based on two data sets: (i) the animal movement database (AMD) and (ii) data from one of the animal transport companies in Switzerland, which we will denote as TRP. Fig 1 provides a graphical representation of characteristics of the data in both data sets. AMD is the national registry for movements of livestock in Switzerland. Since 2011 it is mandatory for all holdings to report all incoming movements of pigs. The FSVO is the data owner while Identitas AG (https://www.identitas.ch/) collects and stores the data. Pig movements in Switzerland are reported at the batch level by the holding receiving the pigs. There is a wide range of different holding types: farms, slaughterhouses, alpine pastures, trading companies, markets, veterinary clinics, and others. Every holding has a unique 7-digit identifier (tvdOrigin and tvdArrival in Fig 1). Some farms may have two or more identifiers if they have holdings at different locations. Apart from the departure and arrival holding, every movement is characterized by the movement date (eventDate), the movement type (movementType, either slaughter or farm-to-farm), and the number of animals in the batch transported (noAnimals). For every holding, we also know the holding type (typeO and typeD), the postal code (postcodeO and postcodeD), the city (cityO and cityD), the canton (cantonO and cantonD), as well as the exact geographic location (latO, longO, latD, and longD). The data also contained information about when movement data was entered in the system (creationDate) and a unique identifier for each movement (id).

The TRP data are organized as tours (tourId) containing a certain number of individual transports that are labelled with sequence numbers (sequenceNumber). A tour contains all the transports operated by a specific truck during a specific period (in most cases 1–2 days). For every individual transport, identifiers of the starting and ending holdings (tvdOrigin and tvdArrival), the exact loading (loadDatetime) and unloading time (dropDatetime, deliveryDate), the number of animals loaded and unloaded (amount), the type of animals transported (species, e.g. piglets, pigs, sows, etc.), and whether or not the truck was cleaned before loading the animals (cleanVehicleRequired) were recorded. The TRP data also contained an identifier for the movement (id) and the batch of animals (batchId), the name of the transport section operating the transport (poolTransportCompany), and whether or not the transport was operated by the customer (consumerDrives).

The period of observation for this study was from 2014 to 2017. The AMD data contained observations from 732,754 transports and the TRP data contained observations from 109,299 transports.

### Data preprocessing

Data preprocessing included removing duplicate entries, self-loops (i.e., movements where the identifiers of the departure and arrival holding are identical), and observations where the involved holdings could not be identified. The reasons for self-loops may have been either misreported transports or transports between holdings of the same farm. After data preprocessing, the AMD data contained information from 722,105 transports. Data preprocessing for the TRP data was similar, but also required removing a small number of transports where loading and unloading time were identical. After data editing, the TRP data contained 106,740 valid entries. Of those entries, 91,613 (85.5%) were present in the AMD data set. In some cases, the date of transport recorded in the TRP data was slightly different from the date in AMD, likely due to misreporting in AMD by the arrival holding. We matched entries in the two data sets based on the week of transport instead of the exact date of transport and found that 94,370 (88.4%) of the transports matched with entries in AMD.

### Network analysis: AMD

In social network analysis terminology, holdings are called nodes or vertices and transports between those holdings are called edges or links [25]. Edges can be either static or dynamic, in which case the edge has a timestamp and is only active at a specific time. Edges can be directed or undirected. In the case of animal transport networks, edges are typically directed.

We created directed temporal networks for every month of the observation period (48 monthly networks) from the AMD data. All edges in these networks were timestamped, i.e. they were only active on the day of transport. The analysis of monthly networks has commonly been justified by asserting that a month roughly corresponds to the “silent spread phase” of a disease [8]. This period was chosen based on reports that numerous infectious diseases in pig herds may remain undetected for 2–4 weeks [26,27]. Depending on the disease modeled, a different period would need to be chosen. For example, the incubation period of the foot-and-mouth disease is typically shorter than a month [28]. However, as we did not intend to model one specific disease in this study, monthly networks were used because they provide a generally accepted estimate of the transmission of a disease within a population before it is detected by disease control authorities and control measures are taken. From an epidemiological point of view, movements to slaughterhouses are considered “dead ends” [9], and we removed these movements as have many previous studies [4,9,15–17].

Network measures that are relevant for disease transmission can be broadly classified into measures of centrality and measures of cohesiveness [2]. Centrality measures are assessed at the node level. Cohesiveness measures indicate how connected the whole network is. We computed the following centrality measures:

- **In- and out-degree**: The in- and out-degree of a node i denotes the number of nodes that are adjacent to, and adjacent from node i, respectively [25]. In our case, the in-degree of a holding is the number of direct animal contacts originating from another holding. Analogously, the out-degree of a holding is the number of direct outgoing contacts. Note that over the period of a month a holding may have multiple contacts with other farms. However, for the in- and out-degree of a holding, a contact with another holding was only counted once because we wanted to measure the centrality of a node.
- **Ingoing contact chain (ICC)**: The ICC was introduced in [9]. It measures the number of holdings that are linked directly or indirectly to a holding through ingoing movements. Importantly, the order of the movements must be taken into account. Note that by considering monthly networks the inter-event time between two consecutive transports is limited to approximately 30 days.
- **Outgoing contact chain (OCC)**: The OCC counts the number of direct or indirect contacts through outgoing movements, taking into account the order of the movements. The OCC is sometimes also called the “set of influence” of a holding [5].

Both ICC and OCC are considered temporal network measures. By taking the temporal sequence of contacts into account, they constitute a more suitable measure of the importance of a holding than conventional (static) measures such as betweenness centrality [25]. Moreover, ICC and OCC may be more suitable in a disease surveillance context than in- and out-degree. For example, consider a holding (A) that only transports pigs to one other holding (B), which has many direct contacts. The out-degree of holding A is one, however, the OCC may be much higher and therefore better represents the risk that this farm may pose for the whole network.

We used the following measures of cohesiveness:

- **Connected components**: A weakly connected component (WCC) is a subgraph in an undirected network in which all nodes are linked directly or indirectly (through a path) to each other. Analogously, a strongly connected component (SCC) is a subgraph in a directed network in which all nodes are linked directly or indirectly to each other [25]. Previous research has suggested using the size of the largest weakly and strongly connected component as the upper and lower bound of potential epidemic size, respectively [8,29].
- **Reachability**: The reachability ratio is the temporal counterpart of the connected components. The reachability ratio is defined as the fraction of node-pairs that are connected through a time-respecting path [5,30].

We also considered one specific monthly network and studied whether the network was scale-free and/or exhibited the small-world property. In scale-free networks, the occurrence of nodes with a comparatively large degree is more common than in random networks. Those nodes can be characterized as hubs and their presence has the effect of making scale-free networks very robust against the random removal of nodes, i.e. they remain connected [31,32]. In scale-free networks, the degrees of nodes follow a so-called power law distribution. We estimated the parameters of such a distribution and assessed the goodness of fit with a log-likelihood ratio test comparing the power law distribution with the log-normal distribution [33,34]. Small-world networks were first introduced by Watts and Strogatz [35] who characterized networks with a small average shortest path length and a large clustering coefficient compared to random networks as small-world networks. A network with small-world properties facilitates the transmission of a disease. In order to test whether the monthly network exhibits small-world properties, we compared its average shortest path (ASP) length and the clustering coefficient (CC) of the largest connected component to the ASP length and the CC of 100 randomly generated networks with the same number of nodes and edges as the monthly transport network [17].

### Network analysis: TRP

**Fig 2:**
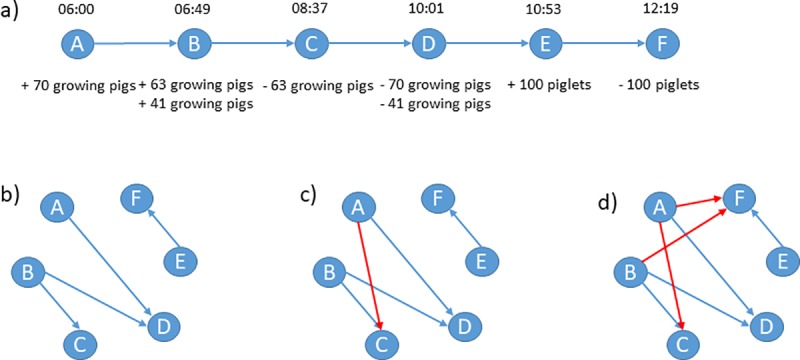
Example of transport data. (a) Time path of the transport data of one tour operated by a single truck. The tour starts at 6.00 a.m. at farm A where 70 pigs are loaded. It then continues to farm B where another 104 pigs are loaded. At farm C, some of the pigs from farm B are unloaded. After that, the truck continues to farm D where the pigs from farm A and the rest of the pigs from farm B are unloaded. The tour ends at 12.19 p.m. with a transport of 100 piglets from farm E to farm F. (b) The network corresponding to the direct transports between farms neglecting the transport sequence. (c) By taking the transport sequence into account, an additional edge between A and C is added because animals from farm A may transmit a disease to C via animals from B. (d) By assuming that the truck can be contaminated and transmit a disease even further, we must include another two edges between A and F and B and F.

We modeled the TRP data similarly to the way we modeled the AMD data. However, individual transports were organized as tours. In order to determine the indirect links due to shared transports, we considered the exact loading and unloading times within a tour. The resulting direct and indirect links between farms were modeled as directed temporal networks. Another approach could have been to create a so called two-mode network [15,25] with nodes being either a holding or a truck. However, many well established network measures are only available for one-mode networks and therefore, we chose to model all networks as one-mode networks. Fig 2 shows one example tour and how direct (b) and indirect edges (c) were extracted from the transport data. Assuming that trucks can be contaminated and act as vectors for transmitting a disease to other animal holdings [4,10,11,13,15,36], we extracted further indirect links between farms (d). As a result, we produced three networks: one representing only edges due to direct transports as reported in the AMD database, one that additionally represents indirect edges due to truck sharing and finally, one that, in addition, contains edges due to the potential contamination of trucks. To assess whether and how the transport data affect the measures of centrality and cohesiveness, we computed the measures described in the previous section for all three networks and compared the results.

### Software

All analysis was conducted in Python 3.6. For the computation of most network measures, we used the library NetworkX (version 1.11). To compute ICC and OCC we developed our own code based on the libraries SciPy (0.19.1) and NumPy (1.15.0).

## Results

### Description of Swiss pig transport network

In order to characterize pig transports in Switzerland, we computed the most frequent types of movements, the average number of pigs per transport, and Euclidean distances of transports. We also investigated inter-cantonal movement patterns, i.e. movements between cantons. Cantons correspond to mostly sovereign political and administrative entities in Switzerland.

Based on AMD, the number of holdings in the network decreased from 11,690 in 2014 to 10,406 in 2017 (Table 1). The results show that there was a steady reduction of continuously producing farms and slaughterhouses over the observation period. Continuously producing farms (76% of all holdings) were by far the most frequent holding type in the network.

**Table 1:** Number of holdings and number of movements in AMD over the observation period (2014–2017). ‘Others’ includes different holding types such as trading companies, markets, and veterinary clinics.

		Year			
		2014	2015	2016	2017
Number of holdings (total)		11,690	11,338	11,120	10,406
	Continuously producing farms	8,923 (76.3%)	8,635 (76.2%)	8,426 (75.8%)	7,885 (75.8%)
	Slaughterhouses	473 (4.0%)	458 (4.0%)	442 (4.0%)	421 (4.0%)
	Others	2,294 (19.6%)	2,245 (19.8%)	2,252 (20.3%)	2,100 (20.2%)
Number of movements (total)		184,148	181,707	179,398	176,852
	Movements to slaughterhouses	133,671 (72.6%)	131,479 (72.4%)	129,266 (72.1%)	127,252 (72.0%)
	Movements to continuously producing farms	40,107 (21.8%)	40,224 (22.1%)	40,124 (22.4%)	39,779 (22.5%)
	Others	10,370 (5.6%)	10,004 (5.5%)	10,008 (5.6%)	9,821 (5.6%)

Table 1 also shows that the number of movements to slaughterhouses steadily decreased over the period of observation while the number of movements to continuously producing farms and other holding types remained relatively stable. A large proportion of movements (72%) were movements to slaughterhouses. As mentioned above, such movements are considered “dead ends” [9] and were removed from the study leaving only 28% of the movements in the Swiss pig transport network relevant for disease transmission.

**Fig 3:**
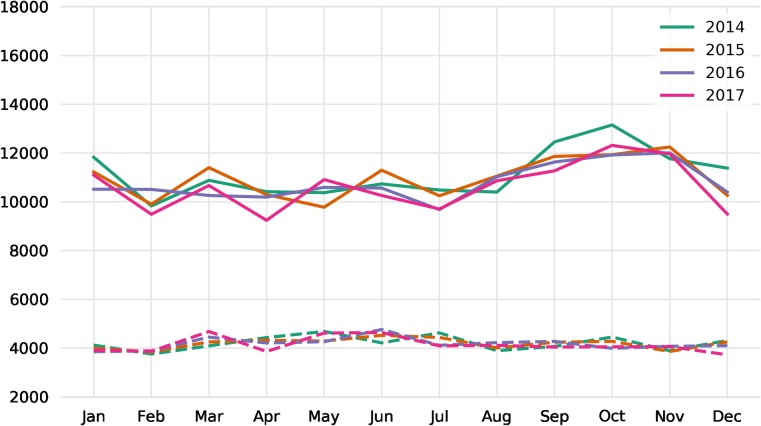
Number of movements per month. Movements to slaughterhouses are plotted with solid lines and movements to holdings other than slaughterhouses are plotted with dashed lines.

The total number of movements to holdings other than slaughterhouses varied only a little, however, there seems to be a seasonal pattern for movements to slaughterhouses (see Fig 3). The greatest number of movements to slaughterhouses for all four years took place in the fall (September–November). We observed a similar pattern for the total number of pigs moved every month (S1 Fig).

A closer look at the AMD data for 2017 revealed that there were 86,648 movements that took place within cantons and 90,204 movements crossing cantonal borders. After excluding movements to slaughterhouses, 27,977 within-canton movements (1,044,599 pigs) and 21,623 between-canton movements (918,041 pigs) remained in the study.

**Fig 4:**
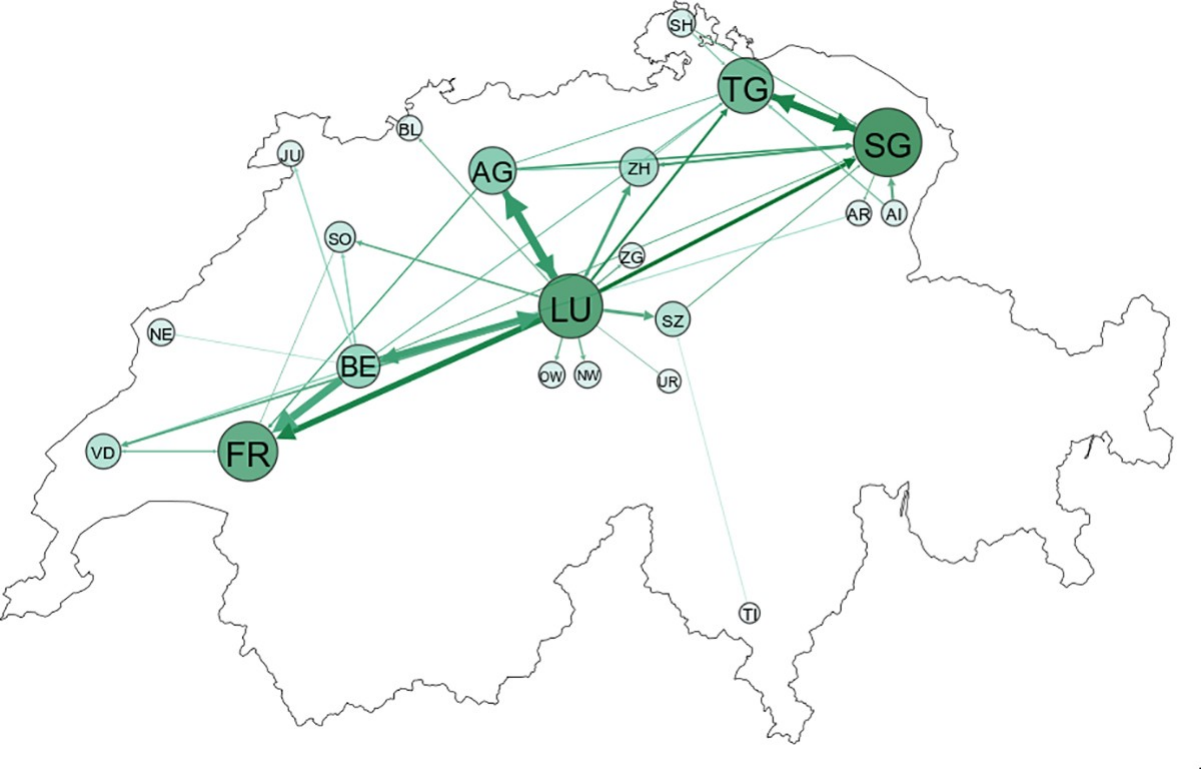
Significant flows of pigs between cantons in 2017. Only flows of 5,000 or more pigs are shown and transports to slaughterhouses are excluded. The size of a node depends on the number of pigs arriving in this canton. St. Gallen (SG) is the largest arrival canton with 131,776 pigs being transported to St. Gallen during the course of 2017. The largest flow of pigs (45,403) occurred between Luzern (LU) and Aargau (AG). The abbreviations for cantons are given in S1 Table.

Fig 4 shows pig flows between cantons in 2017 (excluding movements to slaughterhouses). In order to emphasize significant inter-cantonal relationships, we only plotted flows of 5,000 or more pigs. This figure shows that canton Luzern (LU) played a major role in Swiss pig trade, as the canton had significant trade relations with most other cantons. Important mutual trade patterns also existed between Luzern and Aargau (AG), Luzern and Bern (BE), and Thurgau (TG) and St. Gallen (SG). Fig 4 also shows that canton Fribourg (FR) received a large amount of pigs (107,592 pigs) but at the same time only sent a comparatively small number of pigs (24,442 pigs) to other cantons. Fribourg is home to one of the major slaughter plants in Switzerland and thus the canton may have attracted many fattening farms, leading to the trade pattern described above. The opposite is true for Bern where the number of pigs sent out of the canton (152,337 pigs) was almost 2.5 times greater than the number of pigs received from other cantons (62,133 pigs). A detailed description of the number of incoming and outgoing pigs per canton as well as the numbers of the flows shown in Fig 4 are provided in the supplementary material (S1 and S2 Tables). Note that Switzerland has no major trade patterns with neighboring countries except for with Liechtenstein. In 2017, Switzerland sent 2,229 pigs to Liechtenstein and received 4,192 pigs from Liechtenstein.

The average number of animals per movement was 26 and the median was 15. This indicates that the distribution of the number of animals per movement is right-skewed and that there are outliers. For every transport, we calculated the Euclidean distance. On average, the distance of a transport was 31 km. Ninety percent of all transports were over relatively short distances (up to 76 km). The top 1% of transports covered distances of 131 km or more. The maximum distance travelled was 265 km, which corresponds to a transport from a holding near Geneva to a holding near Chur (Grisons).

The TRP network contained a total of 1,910 holdings. As with AMD, the number of holdings and the number of movements decreased over the observation period (Table 2). Compared with the results from AMD, the TRP data contained more movements between farms and fewer movements to slaughterhouses. However, transports to slaughterhouses were still the dominant type of movement.

**Table 2:** Number of holdings and number of movements in TRP over the observation period (2014–2017). ‘Others’ includes different holding types such as trading companies, markets, and veterinary clinics.

		Year			
		2014	2015	2016	2017
Number of holdings (total)		1,626	1,565	1,511	1,469
	Continuously producing farms	1,318 (81.1%)	1,255 (80.2%)	1,203 (79.6%)	1,174 (79.9%)
	Slaughterhouses	27 (1.7%)	25 (1.6%)	26 (1.7%)	30 (2.0%)
	Others	281 (17.3%)	285 (18.2%)	282 (18.7%)	265 (18.0%)
Number of movements (total)		27,401	26,971	26,435	25,933
	Movements to slaughterhouses	17,048 (62.2%)	16,672 (61.8%)	16,304 (61.7%)	16,120 (62.2%)
	Movements to continuously producing farms	7,927 (28.9%)	7,786 (28.9%)	7,719 (29.2%)	7,563 (29.2%)
	Others	2,426 (8.9%)	2,513 (9.3%)	2,412 (9.1%)	2,250 (8.7%)

As mentioned above, transports were organized as tours of trucks where every tour contained a sequence of individual transports. Overall, there were 33,365 tours of which 6,590 tours contained only one transport of pigs. The average number of transports per tour was 3.2 (median: 3). Ninety percent of tours included 5 or more individual transports with the largest tour containing 16 transports. Note that some tours included transports of other species that were not reported to us (personal communication from the transport company). In most of these transports, the trucks were required to be cleaned before loading a new batch of pigs. It was common for tours to occur at night. In total, there were 3,015 tours that were spread over two days. Most of these tours typically started in the evening of one day and ended in the early morning of the next day. The average transport duration was 157 minutes while the median transport duration was 131 minutes. On average, an individual transport contained 38 pigs (median: 30 pigs). The largest transport contained 500 pigs. Both the average and the median were considerably higher compared to AMD. One possible explanation is that most transports in the TRP data were operated by a transport company whereas the transports reported in AMD include many additional small-scale transports, for example, transports completed by farmers themselves.

### Centrality and cohesiveness in AMD data

The first set of analyses examined the network connectivity and centrality of nodes based on AMD. As the focus of our analysis were monthly networks over a period of four years, we created 48 monthly networks, each representing all movements for one month. The networks were generated as directed multigraphs [25], i.e. networks with potentially multiple links between two specific nodes, as there may be multiple transports on different days between two farms in one month. Note that for computing in- and out-degree, the graphs were transformed to digraphs in order to avoid counting links from or to the same farm multiple times.

On average, the monthly networks consisted of 5,518 nodes and 4,176 edges (3,508 edges in the digraph). Because of removing slaughterhouses from the network, many nodes had no connections to other nodes. There were on average 2,257 nodes with no neighbors in the monthly networks. These nodes were either farms that only reported movements to slaughterhouses in the specific month considered but were otherwise regular farms with in- and out-going movements, or, they were (homebreeding) farrow-to-finish farms that only had movements to slaughterhouses. On average, there were 1,248 farms in the monthly networks that had no in-going movements but at least 1 out-going movement, and 1,568 farms that had only in-going movements but no out-going movements to other farms. Consequently, there were on average only 445 farms with both in- and out-going movements from or to other farms.

Table 3 reports the summary statistics for the maximum in- and out-degree and maximum ICCs and OCCs. Maximum values were reported in order to represent a worst-case scenario with regard to the transmission of a disease. Both the median and mean maximum in-degree were 31, i.e. the farm with the most in-going contacts typically received pigs from 31 different neighbors during the course of a month. Compared to the in-degree, the maximum out-degree was typically lower and ranged between 14 and 22 contacts. By definition, the ICC and OCC for a node must be at least as large as the in- and out-degree, respectively. The average maximum ICC exceeded the average maximum in-degree by 4 contacts. The OCC increased even more compared to the average maximum out-degree: on average, the maximum OCC contained 40 farms.

**Table 3:** Results for centrality and cohesiveness measures over all 48 monthly networks. The networks are based on the animal movement database (AMD).

		Min.	25%	Median	Mean	75%	Max.
Centrality							
	Max. in-degree	25	29	31	31	33	36
	Max. out-degree	14	16	18	18	20	22
	Max. ICC	29	33	34	35	37	45
	Max. OCC	27	35	38	40	44	72
Cohesiveness							
	Size of WCC	1,784 (32.4%)	2,073 (36.4%)	2,138 (39.7%)	2,165 (39.4%)	2,259 (41.9%)	2,460 (47.2%)
	Size of SCC	7 (0.1%)	13 (0.2%)	15 (0.3%)	14 (0.3%)	16 (0.3%)	21 (0.4%)
	Reachability ratio	0.01%	0.01%	0.02%	0.02%	0.02%	0.02%

If we now turn to the results for the cohesiveness of the monthly networks, we can see that the size of the largest weakly connected component ranged between 32.4% and 47.2% of all nodes (Table 3). This means that even in the worst case less than half of all nodes were (weakly) connected. The size of the largest strongly connected component was very small, containing on average 14 nodes (0.3% of all nodes). The reachability ratio shows that typically only 0.02% of all possible node-pairs were connected through time-respecting paths.

**Fig 5:**
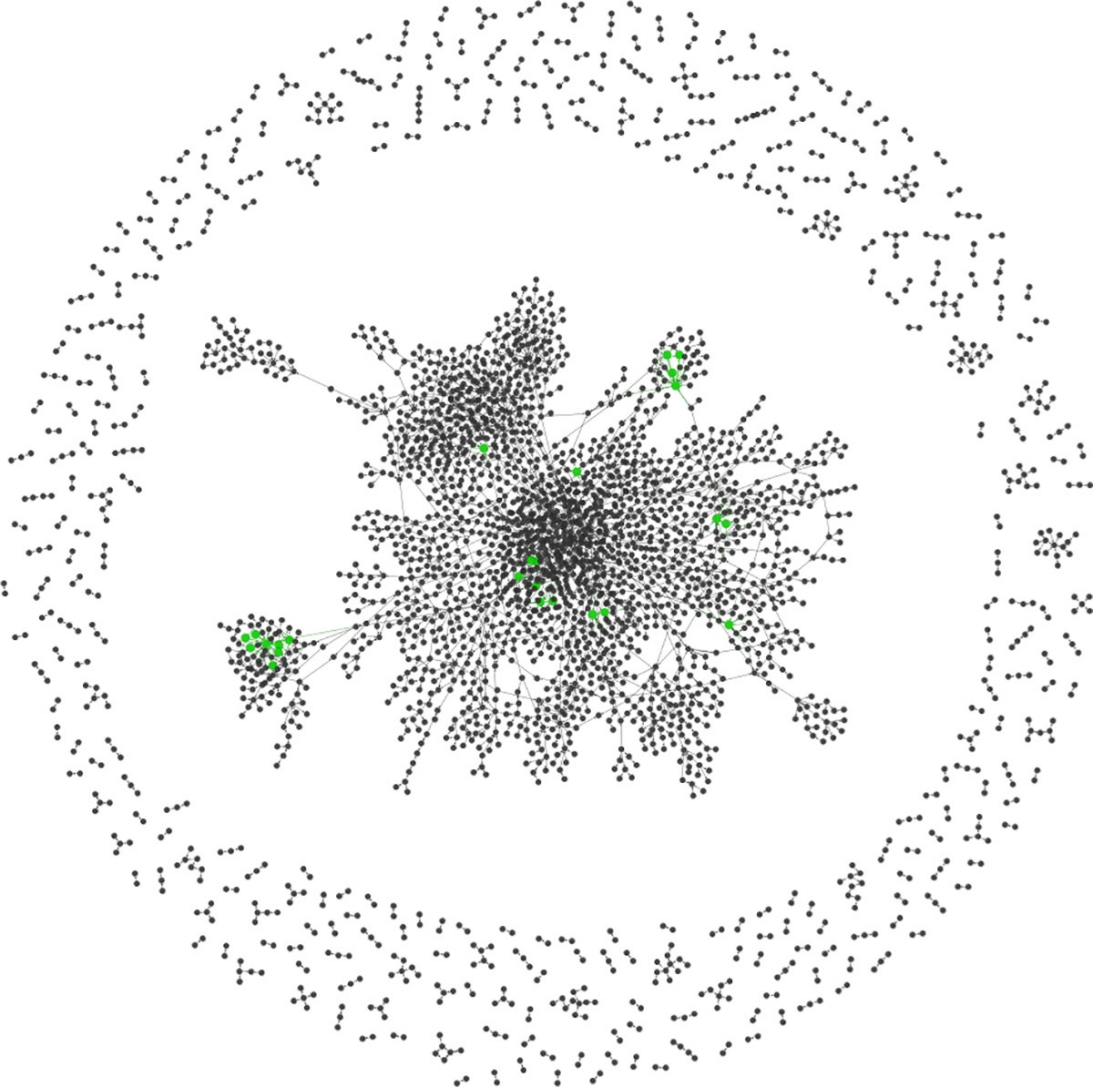
Graph of monthly network in October 2017. Isolated nodes are not shown. The green nodes denote the 25 hubs identified based on the size of ICC and OCC.

In order to show the implications for disease transmission between animal holdings more specifically, we examined one monthly network in detail. As seen above, the activity in the monthly transport networks was typically highest in the fall. Thus, we chose to analyze the movements in October 2017. The results for other monthly networks are similar. The Oct 2017 network contained 5,836 nodes (without slaughterhouses) and 4,048 edges (3,362 edges in the digraph) and was thus a rather sparse network with a density of 0.01%. Density refers to the proportion of actual connections compared to the number of potential connections in the network. Because we removed all slaughterhouses and all edges to slaughterhouses, the network contained 2,781 so-called isolates, i.e. holdings with no connections to other holdings. Fig 5 shows the network without isolates. From the graph, we can see that there is one large (weakly) connected component (2,108 nodes) and many smaller connected components. Only 413 nodes have both a positive in-degree and a positive out-degree and thus are potential hubs in the network.

**Fig 6:**
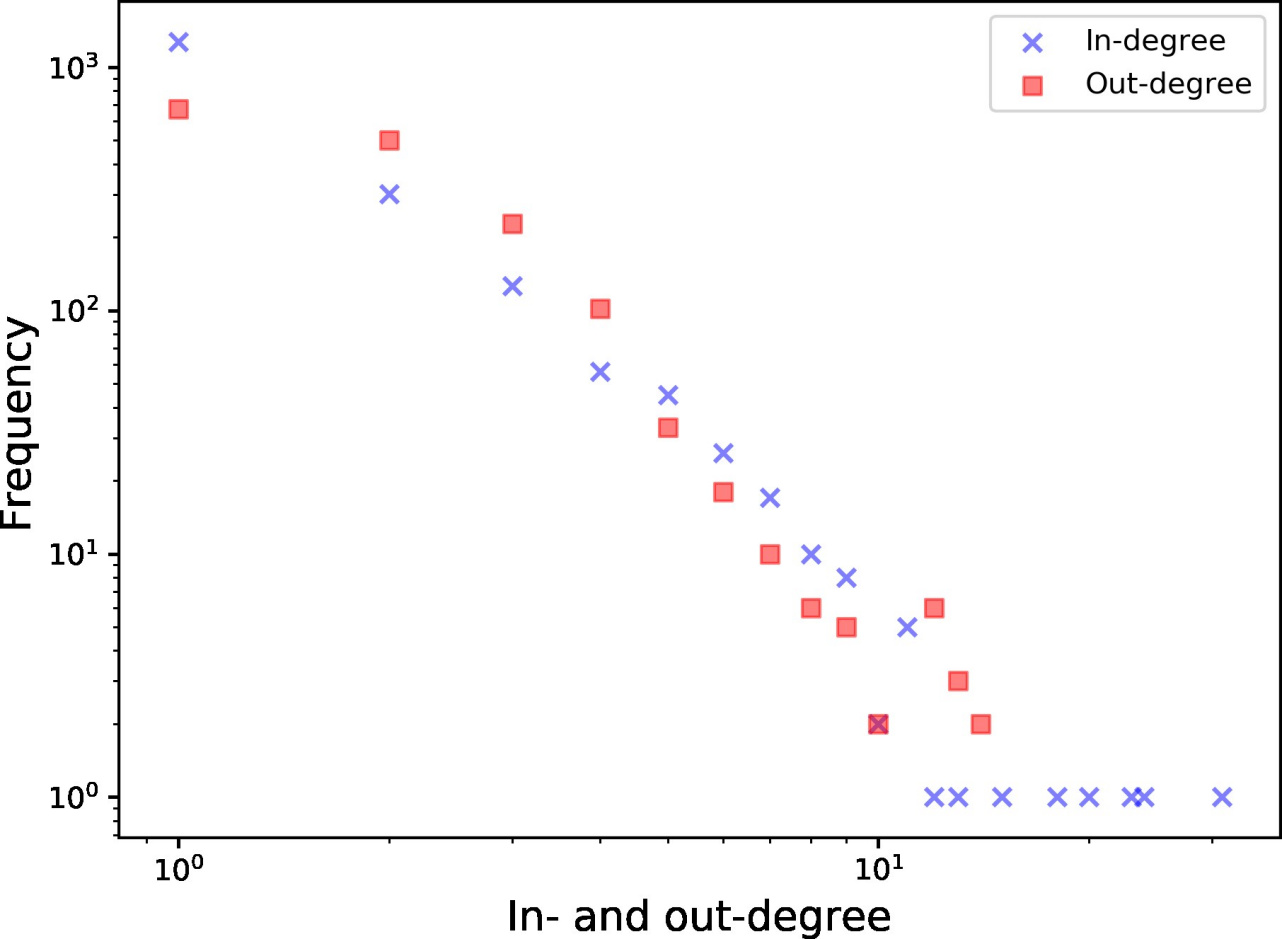
Degree distribution of in-degree (blue crosses) and out-degree (red squares) on a log-log scale for the monthly network of October 2017.

Fig 6 shows the distribution of in-degrees and out-degrees of the nodes in the network. Although the form of the distributions indicates the potential presence of a power law distribution of the degrees, the statistical analysis did not confirm this.

The average shortest path length in the largest connected component was 8.9 and the clustering coefficient was 0.0036. In contrast, the 100 random network realizations exhibited a typical average shortest path length of 31.2 and an average clustering coefficient of 0.0001. Therefore, the network can be characterized as a small-world network.

**Fig 7:**
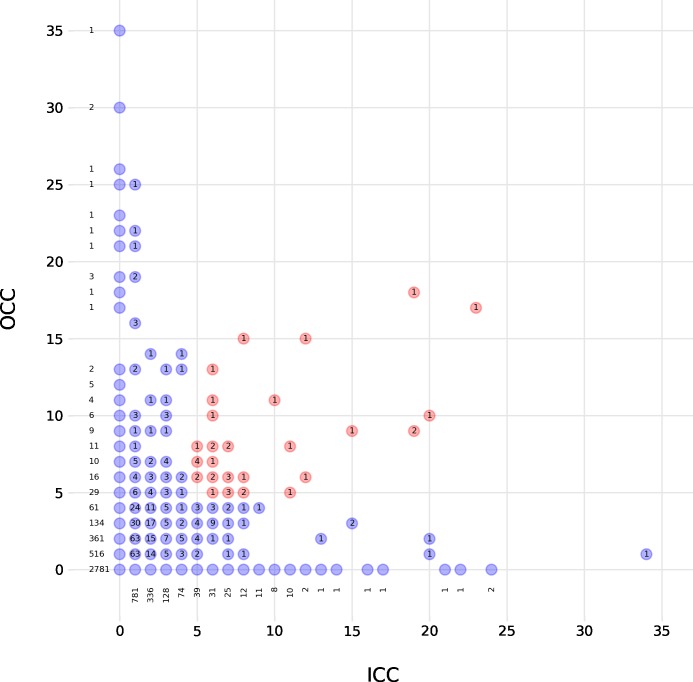
Scatterplot of OCC vs. ICC. The label assigned to every data point indicates the number of nodes (counts) with this combination of OCC and ICC values. For the sake of visibility, some counts are plotted next to the data point. Hubs are highlighted in red.

Based on ICC and OCC values, we identified 25 farms as being crucial for disease transmission (green nodes in Fig 5). These farms exhibit ICC and OCC values larger than 5 and thus may act as hubs (Fig 7). A manual examination of movement patterns of those 25 farms revealed that 15 farms were either farrowing farms (7) or farrowing farms with a nursery (8). The other 10 farms were either piglet producers (3), breeding centers (3) or nursery units (2). For two farms the type could not be identified.

### Centrality and cohesiveness in TRP data

To assess the effect of shared transports and truck contamination on the topology of the contact network, we compared three networks. The first network contained only edges based on direct animal transports between farms (network 1). The second and third network contained additional edges due to shared transports (network 2) and edges due to truck contamination (network 3), respectively. On average, monthly networks for all three types of networks contained 975 nodes. The number of edges, however, varied considerably. On average, the three monthly networks consisted of 793, 912, and 1,648 edges, respectively. If we consider the digraphs, the average number of edges was smaller: 689, 802, and 1,528 edges, respectively.

Table 4 presents the results of the analysis based on TRP. The results for network 1 show that on average the centrality measures were lower than for the networks based on AMD. As mentioned above, the TRP data were only a sample of the full transport network. Thus, the centrality measures computed here could not take into account transports not contained in TRP. The relative size of WCC and SCC and the reachability ratio were higher than in the case of AMD. One possible explanation is that the farms in the TRP sample were more connected than farms in AMD, as they were all customers of the same transport company.

**Table 4:** Results for centrality and cohesiveness measures over all 48 monthly networks. The networks are based on the data from one transport company (TRP).

			Min.	25%	Median	Mean	75%	Max.
**Network 1 (direct)**								
	Centrality							
		Max. in-degree	8	13	19	18	23	35
		Max. out-degree	11	15	17	17	19	24
		Max. ICC	9	16	21	22	25	46
		Max. OCC	13	22	26	26	30	38
	Cohesiveness							
		Size of WCC	331 (35.4%)	465 (47.1%)	483 (49.9%)	484 (49.6%)	521 (53.8%)	578 (58.5%)
		Size of SCC	4 (0.4%)	5 (0.5%)	5 (0.5%)	6 (0.6%)	6 (0.7%)	8 (0.8%)
		Reachability ratio	0.07%	0.08%	0.09%	0.09%	0.09%	0.11%
**Network 2 (shared truck)**								
	Centrality							
		Max. in-degree	9	15	19	20	24	37
		Max. out-degree	15	19	21	22	24	31
		Max. ICC	10	19	24	25	31	50
		Max. OCC	20	30	32	33	37	42
	Cohesiveness							
		Size of WCC	367 (40.2%)	529 (54.0%)	557 (57.3%)	551 (56.4%)	583 (59.6%)	659 (63.4%)
		Size of SCC	4 (0.4%)	5 (0.5%)	5 (0.6%)	6 (0.6%)	7 (0.7%)	9 (0.9%)
		Reachability ratio	0.08%	0.10%	0.10%	0.10%	0.11%	0.13%
**Network 3 (contaminated truck)**								
	Centrality							
		Max. in-degree	14	20	25	25	30	50
		Max. out-degree	20	25	28	28	31	34
		Max. ICC	24	36	43	44	49	81
		Max. OCC	36	48	53	52	57	70
	Cohesiveness							
		Size of WCC	675 (74.5%)	738 (76.7%)	765 (78.2%)	766 (78.5%)	799 (79.8%)	860 (84.0%)
		Size of SCC	5 (0.5%)	7 (0.7%)	9 (0.9%)	9 (0.9%)	11 (1.2%)	17 (1.8%)
		Reachability ratio	0.20%	0.22%	0.24%	0.24%	0.25%	0.34%

As seen in Table 4, the cohesiveness of the network increased slightly by considering additional indirect links due to truck sharing (network 2). For example, the average size of the largest WCC increased from 49.6% to 56.4%. At the same time, all centrality measures were between 2 and 7 nodes larger on average. By considering all links including additional links due to truck contamination (network 3), we observed a significant increase in the connectivity for the whole network. The size of the largest WCC, for example, contained 78.5% of all nodes compared to 56.4% for network 2. Accordingly, the reachability ratio increased from 0.09% in network 1 to 0.24% in network 3. The consideration of links due to truck contamination also had a strong effect on the centrality measures, especially on ICC and OCC. The average values of the maximum ICC and OCC were 44 and 52 farms, respectively. In an absolute worst-case scenario, a farm could potentially infect 70 other farms in this sample, which is almost twice as many farms as in network 1.

## Discussion

The main objective of our study was to assess the effect of the topology of the Swiss pig transport network on the potential for disease transmission between animal holdings. The analysis was based on (i) the official animal movement database in Switzerland, and (ii) a sample of transport data from one animal transport company in Switzerland. The sample covered approximately 13% of the transports reported in AMD and the data comprised transports in all major regions of Switzerland. The present study was designed primarily to compare the topology of networks based on (i) to networks based on (ii). We examined the effect of the structure of the Swiss pig industry, which differs from other pig industries in Europe because of the high number of small holdings, the frequent trade of small batches of pigs and the use of vehicles that are not always properly cleaned and disinfected. For both data sources, we restricted our analysis to monthly networks. As explained earlier, movements to slaughterhouses were not considered in the network analysis.

A detailed analysis of trade flows between cantons (44% of all farm-to-farm movements) revealed the crucial role of the canton Luzern. It had significant trading patterns with most other cantons, as it was the origin for nearly 30% of all pigs sent to other cantons, and received 13% of all pigs traded between cantons. For disease surveillance, this implies that the surveillance of highly connected farms in Luzern is crucial. If an outbreak occurred in Luzern, a disease could quickly transmit to many other cantons of Switzerland if it was undetected. Disease surveillance should also focus on farms in the canton Bern as this canton had the second largest number of pigs sent to other cantons. Due to the geographical characteristics and the small size of the country, the average Euclidean distance travelled was relatively small (31 km). In fact, 90% of all movements covered a distance of 76 km or less. This is similar to results found for British pig movements [17], whereas French pig movements typically cover much longer distances [15]. In some cases (e.g. mountainous regions), the Euclidean distance may not be a good measure for the actual distance travelled. However, as can be seen in Fig 4, Swiss pig trade predominantly occurred in the Swiss Plateau, which denotes the region between Lake Geneva and Lake Constance [37]. There is only little pig trade across the Swiss Alps and the Euclidean distance may thus be a plausible estimate of the distance travelled for most transports. However, as the analysis of the TRP data has shown, transports are often part of a tour where a truck first loads animals from a few different farms before it arrives at the first unloading place. Hence, the distance travelled might actually be larger in reality than the AMD data suggests. Importantly, the analysis of the TRP data also showed that a tour often includes transports of species other than pigs. Although a truck is usually required to be cleaned after the transport of another species, this might pose an additional risk for cross-species transmission of diseases.

The AMD networks were very sparse, having a density of 0.01%. As a result of removing the slaughterhouses, almost 41% of the holdings contained in the network were isolated holdings with no connections to other farms. This indicates a high degree of fragmentation that should prevent a disease from transmitting to large parts of the network [2]. On average, there were only 445 holdings (8%) with in- and outgoing contacts to other farms. Theses holdings are especially important for disease surveillance as they constitute the pool of potential hubs, i.e. farms with a high in- and out-degree compared to the other farms [2]. Such farms may act as “super-spreaders” [17] and should be specifically targeted for disease surveillance.

SCC and WCC have been proposed as estimates for the lower and upper limit of the outbreak size, respectively [2,8]. The comparison of these two measures with one possible temporal counterpart (reachability ratio) shows that the static measures (SCC and WCC) overestimate the outbreak risk. In fact, only 0.02% of all possible node pairs in monthly networks were connected through a time-respecting path. This agrees with our observation that the Swiss pig transport network is highly fragmented. To determine the maximum outbreak size in a worst-case scenario, we propose to use the maximum OCC as has been suggested in [16]. Thus, for the Swiss pig industry we expect a worst-case outbreak size of 70 to 80 holdings. Interestingly, a Swedish study found considerably higher values for the maximum OCC (and ICC) in monthly networks [9], which indicates a smaller degree of fragmentation of the Swedish network. A possible explanation for this might be the pyramidal structure of the Swedish pig industry [9] which is fundamentally different from the highly decentralized Swiss pig industry. Overall, our study strongly supports the findings of previous studies which advocate for the use of temporal network measures, especially ICC and OCC in the context of disease surveillance and control [8,9,14,16].

The analysis of one specific monthly network (October 2017) revealed several interesting results. First, in contrast to other studies [15,17], we did not find evidence for a scale-free network. Consequently, nodes with a large in- and out-degree compared to the average degree (hubs) are not very common. In this study, we identified hubs based on ICC and OCC values instead of in- and out-degree. As expected, we found relatively few farms fulfilling this criterion (25 farms). These farms were identified as farrowing farms, piglet producers, breeding centers, or nursery units. Interestingly, 10 hubs had an in-degree of 1, and 5 of them had an ICC value that was greater than or equal to 15. It is important to note that most of the hubs with a low in-degree and a large ICC were connected to other hubs thereby ‘inheriting’ part of their connectedness from these hubs. Second, Fig 7 shows that there was a number of farms with very high OCC values and zero ICC values. While these nodes do not count as hubs, they are important for disease surveillance, as they constitute source nodes that can transmit a disease to many other holdings. Analogously, farms with high ICC values and zero OCC values exhibit a high risk of being infected (super-receivers). These findings suggest targeting three groups of farms in a disease surveillance system: 1) hubs, 2) high OCC-farms, and 3) high ICC-farms. Finally, the network exhibited small-world properties. However, both the clustering coefficient and the average shortest path length did not take the temporal nature of the transport network into account. Thus, we question the small-world property’s ability to explain the transmission of a disease in this context. Further work is required to establish the effect of small-world characteristics in temporal networks.

One of the main objectives of this study was to assess the effect of shared transports and truck contamination on the topology of the contact network based on TRP. What is striking about the results for the comparison of the three different networks is the large increase in connectivity due to the additional consideration of truck contamination while the effect of adding edges due to truck sharing was only moderate. This finding suggests special attention is needed for the control of infectious diseases that are mainly transmitted by fecal-oral routes (e.g. Brachyspira spp., Salmonella spp., etc.) and other infections that require close contact of infected animals (e.g. Actinobacillus pleuropneumoniae, toxigenic Pasteurella multocida, etc.). However, in the case of infections that can also be airborne (e.g. swine influenza virus, porcine reproductive and respiratory syndrome virus, etc.) this may be less relevant. Considering all indirect edges due to truck sharing and contamination increased the size of the largest WCC by 58.3%. Similarly, the reachability ratio was more than 2.5 times larger compared to the network with only direct edges. These findings are consistent with previous studies that examine the effect of truck sharing and contamination on the topology of the network [13,15]. Therefore, we can surmise that the consideration of additional, indirect ways of disease transmission will have a substantial effect on the topology of the network and may thus influence how quickly and how far a disease can transmit. Since the TRP data are only a sample of transport data, the ICC and OCC values may in reality be even larger.

Together these results provide important insights for the design of disease surveillance and control strategies. The Swiss pig industry is more fragmented than pig production systems in other countries, which makes large outbreaks of infectious diseases unlikely. Static network measures suffer from some serious drawbacks. Most importantly, they tend to overestimate the overall risk of disease transmission in a network. Addressing the problem with methods from temporal network analysis allows us to compute more realistic individual and overall risk measures. Finally, the comparison of networks based on AMD and TRP shows that the additional consideration of truck sharing and contamination as indirect ways of disease transmission have a large effect on the topology of the network. Hence, conclusions based on conventional animal movement data such as AMD may underestimate the actual risk of disease transmission between animal holdings.

## Supporting information

S1 Fig Number of pigs transported every month. Pigs transported to slaughterhouses are plotted with solid lines and pigs transported to holdings other than slaughterhouses are plotted with dashed lines.
(TIF)

S1 Table Number of pigs traded with other cantons or traded within the canton in 2017. The table additionally includes the abbreviation for every canton.
(PDF)

S2 Table Number of pigs moved between cantons in 2017. Only flows of 5,000 pigs or more are listed. Source denotes the canton of origin and Target denotes the canton of arrival.
(PDF)
