# Supplementary figures and images for: The pig transport network in Switzerland: Structure, patterns, and implications for the transmission of infectious diseases between animal holdings
Source: PLoS One. 2019 May 31;14(5):e0217974. doi: 10.1371/journal.pone.0217974 (PMC6544307; doi:10.1371/journal.pone.0217974)

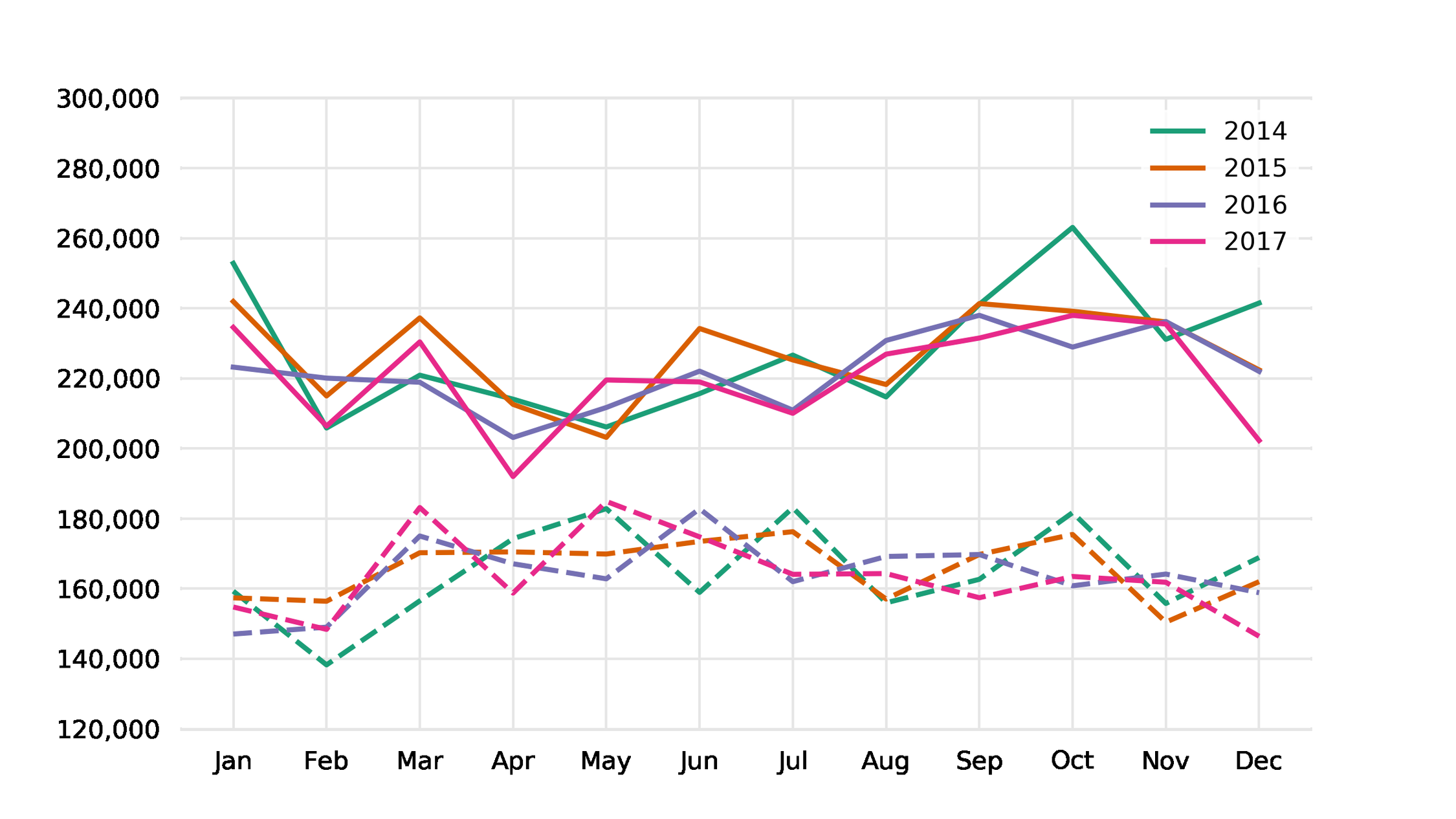

Supplement: S1 Fig — Pigs transported to slaughterhouses are plotted with solid lines and pigs transported to holdings other than slaughterhouses are plotted with dashed lines. (TIF) [file pone.0217974.s001.tif]
