# Supplementary material for: The pig transport network in Switzerland: Structure, patterns, and implications for the transmission of infectious diseases between animal holdings
Source: PLoS One. 2019 May 31;14(5):e0217974. doi: 10.1371/journal.pone.0217974 (PMC6544307; doi:10.1371/journal.pone.0217974)
Supplement: S1 Table — The table additionally includes the abbreviation for every canton. (PDF) [file pone.0217974.s002.pdf]

Abbr.	Canton	Pigs received from other cantons	Pigs sent to other cantons	Pigs traded within canton
AG	Aargau	73,740	85,852	42,458
AI	Appenzell Innerrhoden	14,281	31,409	9,220
AR	Appenzell Ausserrhoden	13,005	19,022	5,283
BE	Bern	62,133	152,337	204,782
BL	Basel-Landschaft	13,137	9,961	447
BS	Basel-Stadt	140	-	-
FL	Fürstentum Liechtenstein	1,976	431	-
FR	Fribourg	107,592	24,422	32,899
GE	Geneva	4,011	70	23
GL	Glarus	5,064	58	221
GR	Grisons	8,334	998	3,083
JU	Jura	14,657	8,521	8,182
LU	Luzern	119,837	272,822	459,498
NE	Neuchâtel	17,785	5,407	2,070
NW	Nidwalden	16,529	4,291	2,637
OW	Obwalden	15,747	5,852	1,203
SG	St. Gallen	131,776	82,714	96,614
SH	Schaffhausen	20,249	20,343	3,488
SO	Solothurn	27,999	25,931	7,074
SZ	Schwyz	39,247	21,034	3,090
TG	Thurgau	96,863	61,049	120,184
TI	Ticino	135	8,259	239
UR	Uri	8,810	686	184
VD	Vaud	39,755	10,898	27,352
VS	Valais	1,868	43	39
ZG	Zug	13,340	18,292	8,561
ZH	Zürich	50,031	47,339	5,768
