# Supplementary material for: The pig transport network in Switzerland: Structure, patterns, and implications for the transmission of infectious diseases between animal holdings
Source: PLoS One. 2019 May 31;14(5):e0217974. doi: 10.1371/journal.pone.0217974 (PMC6544307; doi:10.1371/journal.pone.0217974)
Supplement: S2 Table — Only flows of 5,000 pigs or more are listed. Source denotes the canton of origin and Target denotes the canton of arrival. (PDF) [file pone.0217974.s003.pdf]

Source	Target	No. of animals	Source	Target	No. of animals
AG	FR	9,209	SG	AI	6,108
AG	LU	38,352	SG	AR	7,152
AG	SG	11,414	SG	TG	37,479
AG	TG	6,121	SG	ZH	10,026
AG	ZH	6,966	SH	SG	7,541
AI	SG	15,820	SH	TG	8,347
AI	TG	9,660	SO	BE	6,841
AR	BE	8,241	SO	FR	5,222
BE	FR	43,424	SO	LU	5,901
BE	JU	10,706	SZ	SG	6,941
BE	LU	37,915	TG	AI	5,549
BE	NE	5,277	TG	SG	36,743
BE	SG	6,204	TI	SZ	7,013
BE	SO	10,001	VD	FR	7,860
BE	TG	7,011	ZG	LU	6,887
BE	VD	10,100	ZH	AG	6,522
FR	BE	7,802	ZH	LU	8,987
FR	VD	7,700	ZH	SG	13,668
LU	AG	45,403	ZH	TG	6,064
LU	BE	31,553			
LU	BL	8,445			
LU	FR	33,733			
LU	NW	11,084			
LU	OW	10,547			
LU	SG	22,401			
LU	SO	11,814			
LU	SZ	19,605			
LU	TG	14,940			
LU	UR	5,603			
LU	VD	13,258			
LU	ZG	8,687			
LU	ZH	18,610			
